# In Situ Growth of Lanthanide Coordination Polymers on Oxide Glass and Optical Fibers: A Promising Material for Chemical Sensing

**DOI:** 10.1021/acsami.5c16933

**Published:** 2025-12-17

**Authors:** Renato G. Capelo, Francis D. R. Garcia, Clément Strutynski, Frédéric Désévédavy, Gregory Gadret, Caroline M. da Silva, Guilherme Arroyos, Regina C. G. Frem, Guillermo Orellana, Frédéric Smektala, Danilo Manzani

**Affiliations:** † São Carlos Institute of Chemistry, University of São Paulo, 13566-590 São Carlos, São Paulo, Brazil; ‡ Laboratoire Interdisciplinaire Carnot de Bourgogne ICB UMR 6303, Université Bourgogne Europe, CNRS, 21000 Dijon, France; § Institute of Chemistry, São Paulo State University, 14800-900 Araraquara, São Paulo, Brazil; ∥ Facultad de Ciencias Químicas, 16734 Universidad Complutense de Madrid, 28040 Madrid, Spain

**Keywords:** luminescence, lanthanide-coordination polymers, oxide glasses, optical fibers, chemical sensing

## Abstract

Coordination polymers (CPs) have gained significant attention as chemical sensors due to their highly tunable porous structures, enabling selective interactions with target analytes. Lanthanide-based coordination polymers (Ln-CPs) have been extensively utilized in optical sensing, owing to their photoluminescent properties. However, these applications typically require deposition on stable substrates with the appropriate chemical and physical characteristics. This study introduces a simple and rapid *in situ* synthesis and coating process for Ln-CPs on oxide glass bulks and optical fibers. Eu^3+^-based CPs were successfully coated onto tellurite and phosphate glasses by using polycarboxylic acids as ligands. Although slight deviations from previously reported crystalline structures were observed, luminescent coatings were effectively formed and demonstrated good adhesion to the tellurite glass substrates. These materials exhibited potential selectivity toward carbonyl compounds, showing an enhanced luminescent response at low concentrations. The successful integration of Ln-CPs onto TZN-based optical fibers underscores their potential for real-time remote sensing, offering promising applications in environmental monitoring, industrial safety, and biomedical diagnostics.

## Graphical abstract

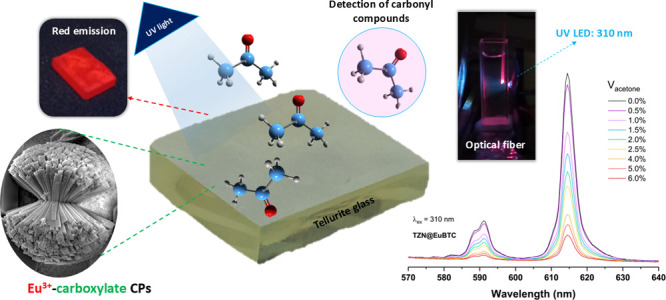

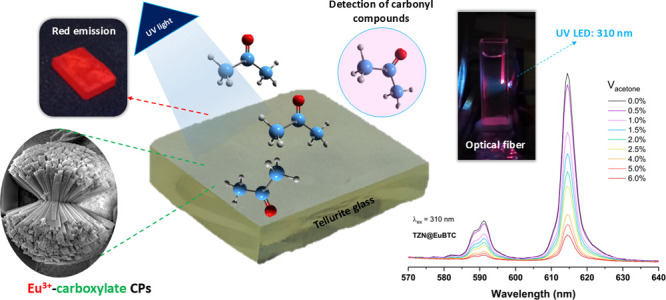

## 1 Introduction

Optical sensors can provide faster response times, greater selectivity, and enhanced detection capabilities compared to semiconductor or electrochemical sensors, while also enabling real-time detection without degradation of the sample.^1^ However, some sensing platforms require chemoresponsive agents to allow interactions between the evanescent field of the waveguides and the analyte, generating optical responses.^2,3^ In this context, coordination polymers (CPs), an advanced class of materials formed by the self-assembly of metal ions or clusters with organic linkers,^4^ represent a promising class of materials for chemical sensing.^5–7^

CPs offer versatile functionality by combining the properties of their metal and organic components, enabling a wide range of applications.^8–12^ CPs containing lanthanide ions are particularly noteworthy due to the unique electronic, magnetic, and optical properties arising from their shielded 4f electrons.^13,14^ In this regard, these Ln-CPs exhibit exceptional luminescent properties, making them suitable candidates for applications such as light-emitting diodes (LEDs), biomedical imaging, and optical sensing.^15–19^

Emission from Ln-CPs typically occurs by the*antenna effect* of the ligands, which absorb light and, assisted by vibronic coupling, transfer the excited state energy to the metal, resulting in lanthanide ion luminescence.^20,21^ Optical sensing with these materials is based on changes in the luminescence intensity when analytes interact with the CP, disturbing the energy transfer from the excited state of the ligands to the emitting center.^22,23^ For instance, Eu^3+^-based Ln-CPs have been extensively studied using carboxylate ligands to form 3D networks with large pore volumes and high surface areas while also exploring the *antenna effect* as a sensing mechanism.^24,25^ The porous nature of Eu-carboxylate CPs allows for the selective uptake of guest molecules, which can modulate their luminescence by either quenching or enhancing the emission from Eu^3+^ ^26,27^

For practical sensing applications, CPs must maintain stability when exposed to the chemical species of interest, typically by forming a film over an appropriate substrate.^28,29^ Furthermore, an adequate supporting material is also required to facilitate the transmission of the excitation and emitted light after the interaction with an analyte. In this regard, oxide glasses with transparency windows from ultraviolet (UV) to visible ranges can act as suitable host substrates for Ln-CPs.^30–32^ Additionally, optical fibers fabricated from these glasses represent a potential host material for Ln-CPs, particularly in applications involving fiber-optic chemical sensing.^22,33^

In this study, we aim to investigate the *in situ* growth of Ln-CPs on the surfaces of tellurite and phosphate glasses as well as optical fibers, exploring their potential for chemical sensing applications. Specifically, we report the synthesis of a series of glass@Ln-CP composites that combine glassy substrates and Eu^3+^-based CPs containing carboxylate ligands. The composites were obtained through a simple and rapid procedure, and their photoluminescent properties were examined for the detection of small organic molecules.

## 2 Experimental Section

### 2.1 Materials and Methods

All chemicals were purchased from commercial sources and used without prior treatment. The glass samples were synthesized by a conventional melt-quenching method from high-purity tellurium oxide (TeO_2_, Aldrich 99.99%), zinc oxide (ZnO, Aldrich 99.9%), anhydrous zinc fluoride (ZnF_2_, Aldrich 99.9%), and anhydrous sodium carbonate (Na_2_CO_3_, Aldrich 99%). The Ln-CP precursors, 1,3,5-benzenetricarboxylic acid (H_3_BTC), 2,6-pyridinedicarboxylic acid (H_2_PDC), 1,2,4,5-benzenetetracarboxylic acid (H_4_B4C), and EuCl_3_.6H_2_O, were purchased from Sigma-Aldrich. The composites were characterized by Raman spectroscopy, X-ray diffractometry (XRD), scanning electron microscopy using a field emission gun (FEG-SEM), and photoluminescence (PL) spectroscopy to evaluate the formation of Ln-CPs on glass surfaces.

### 2.2 Glass Substrate Synthesis

The glass samples were prepared by a conventional melt-quenching process, and the compositions and labels are shown in Table 1. For the tellurite glasses (TZN), the batch was heated in a furnace in the temperature range of 700–750 °C for 20 min in a gold crucible, then poured into a preheated brass mold, annealed for 120 min at 250 °C, and finally cooled at the rate of 10 °C.min^–1^ down to room temperature. In the case of the aluminum-phosphate matrix obtained with phosphoric acid as the phosphorus precursor (PNKA), the melting process was carried out at 1100 °C for 40 min and then poured into a brass mold. The mold was preheated at 360 °C, and the annealing was performed at 400 °C for 5 h, followed by a cooling process at the rate of 5 °C. The samples were cut and polished before the coating process.

**Table 1:** Labels and Nominal Molar Concentrations of the Glass Substrates

	nominal molar composition (mol %)						
sample label	TeO_2_	ZnO	ZnF_2_	Na_2_O	P_2_O_5_	K_2_O	Al_2_O_3_
TZN	75	15	0	10			
TZNF15	75	0	15	10			
TN	90	-	-	10			
PNKA	-	-	-	20	45	20	15

The TZN glass preform for fiber drawing was prepared following the same procedure described above but using a cylindrical mold and obtaining a preform measuring 5 cm in length with a diameter of 16 mm. Subsequently, the preform was drawn into the fiber under a He gas flow, on a commercial Control Interface Limited drawing tower at approximately 400 °C with a loading rate of 1.0 mm min^–1^. Similarly, the PNKA cylindrical preform was fabricated using the melt-quenching technique, and optical fibers were drawn under analogous conditions, except for a higher temperature of around 500 °C. Both resulting single-index optical fibers exhibited diameters ranging from 250 μm to 1 mm.

### 2.3 *In Situ* Growth of Ln-CPs on Glass and Optical Fiber Surfaces

The glass substrate surfaces were initially rinsed with isopropyl alcohol and acetone. For the *in situ* growth of Ln-CPs, two methods were used: microwave-assisted hydrothermal synthesis and hot-solution-assisted synthesis. CP coatings were initially prepared through microwave-assisted hydrothermal synthesis by using a Teflon cylinder reactor at a temperature of 180 °C. Following the addition of Ln-CP precursors into the reactor chamber, the optical glass pieces were immersed in the reactive medium. Similarly, hot-solution-assisted synthesis of Ln-CPs was conducted in an open system, utilizing a hot plate set to 90 °C for durations ranging from 5 to 20 min with continuous stirring. This streamlined approach not only simplifies the synthesis conditions but also facilitates the application of Ln-CP coatings onto optical fibers, immersing the fiber end (approximately 2–5 cm) into the solution. Specific procedures corresponding to the synthesis of each distinct Ln-CP were meticulously adhered to.

#### 2.3.1 Synthesis of {[Eu­(BTC)­(PDC)]_*n*_} CP (**1**)

This synthesis follows the procedure described by Da Silva et al.,^34^ using two ligands containing carboxylate groups: H_3_BTC and H_2_PDC. H_3_BTC (0.40 mmol, 84 mg) and 10 mL of deionized water were added to the reactor, and the pH was adjusted to 3.6 with NaOH solution (0.5 mol. L^–1^). Then, H_2_PDC (1.20 mmol, 200 mg) and EuCl_3_.6H_2_O (0.28 mmol, 100 mg) were added, and the mixture was heated for 20 min and cooled to room temperature. The same procedure was performed with a synthesis time of 10 min.

#### 2.3.2 Synthesis of {[Eu­(BTC)]_*n*_} CP (**2**)

This CP was obtained using the same procedure described above, removing the H_2_PDC ligand. Then, H_3_BTC (0.40 mmol, 84 mg) was added to 10 mL of deionized water, and the pH was adjusted to 3.6. The same amount of EuCl_3_.H_2_O (0.28 mmol, 100 mg) was added to the Teflon reactor, and the mixture was heated for 20 min.

#### 2.3.3 Synthesis of {[Eu­(B4C)]_*n*_} CP (**3**)

This compound was synthesized by following a similar procedure to that used for Da Silva et al.,^34^ adapting some parameters based on procedures reported in the literature.^35,36^ H_4_B4C (0.40 mmol, 90 mg) was dissolved in 10 mL of deionized water, and the pH was adjusted to 5.0 using an aqueous solution of NaOH (0.5 mol L^–1^). After the EuCl_3_·6H_2_O addition (0.28 mmol, 100 mg), the reactor was sealed, heated for 20 min, and then cooled to room temperature.

### 2.4 Characterization Techniques

#### 2.4.1 FEG-SEM

A JEOL JSM-7200F operating at a beam energy of 15 kV was used to obtain the FEG-SEM. The glass samples coated with Ln-CPs were analyzed by using gold as a support, and high-resolution images of the glass-CP interface and the Ln-CP surfaces were taken.

#### 2.4.2 X-ray Diffractometry

XRD measurements were performed using a Siemens D 5000 diffractometer at room temperature with a wavelength corresponding to the K_α1_ layer of Cu (1.54056 Å). The crystallographic base ICSD (Inorganic Structure Database) was used, which presents an updated catalog of crystallographic phases already described.

#### 2.4.3 PL Spectroscopy

PL spectroscopy measurements were performed by a Horiba Fluorolog-3 FL-1050 spectrofluorometer for the glasses coated with Ln-CPs. Luminescence spectra were recorded at room temperature from 400 to 700 nm, under excitation wavelengths varying from 300 to 395 nm and using slit widths between 1 and 5 nm, depending on the sample. The data were collected at every nanometer, with an integration time of 0.1 s for each step. In addition, decay time measurements were conducted in an Agilent Cary Eclipse spectrofluorometer in the phosphorescence mode, monitoring the main emission peak centered at 616 nm, under excitation at 300 nm.

#### 2.4.4 PL Measurements on Optical Fibers

PL measurements on coated optical fibers were performed using samples with lengths ranging from 7 to 15 cm and diameters between 250 μm and 1 mm. The Ln-CP coatings were excited using a UV lamp (254 nm) or a Roithner Laser Technik DUV310-SD3535EN 308 nm, 43 mW (@350 mA), 35° quartz-domed SMD LED. Emission was collected at the opposite tip of the optical fiber with a portable Hamamatsu C-13555MA-5345 fiber optic spectrometer. An integration time of 0.2 s was employed, with the accumulation of 100 spectra for improved signal-to-noise ratios.

#### 2.4.5 Chemical Sensing Trials

The PL measurements for the sensing tests were carried out under the same conditions described in Section 2.4.3 for bulk samples and Section 2.4.4 for fiber samples. The samples were immersed in a quartz cuvette containing aqueous solutions of various organic compounds, with concentrations ranging from 0.05% to 6% by volume. The initial volume of the solution was 3 mL. Each sample was tested three times (*n* = 3) across all of the experiments. The data were subjected to statistical analysis, with results reported as the mean ± standard deviation (SD).

## 3 Results and Discussion

### 3.1 Adhesion, Morphology, and Structural Characterization

The glass compositions of the TZN system were initially used for the *in situ* growth of Ln-CPs, demonstrating good surface adhesion. This glass system, particularly those containing ZnF_2_, had been previously studied and exhibited a wide transparency range (from UV to mid-infrared) and good thermal stability.^37^ A composition without Zn (TN) was also tested to evaluate the influence of this transition metal on the coating capability of the glass. Additionally, the PNKA matrix was used as a substrate for Ln-CP growth due to its broader transparency in the UV region than found in tellurites,^38^ but the resulting coatings were less homogeneous and appeared to have weaker adhesion compared to those on TZN substrates. The composites produced in this study are described in Table 2, which indicates the carboxylate ligand coordinating to Eu^3+^, the glass composition of the substrate, and its form, either as a monolith (glass bulk) or as a single-index optical fiber (OF).

**Table 2:** Composite Samples Synthesized with a CP Containing Eu^3+^ Coated on Glass Substrates

	ligand precursor				
sample label	H_3_BTC	H_2_PDC	H_4_B4C	glass matrix	composite form
TZN@[Eu(BTC)(PDC)]	X	X		TZN	bulk
TZNF15@[Eu(BTC)(PDC)]	X	X		TZNF15	bulk
TZN@EuBTC	X			TZN	bulk/OF
TZNF15@EuBTC	X			TZNF15	bulk
TN@EuBTC	X			TN	bulk
PNKA@EuBTC	X			PNKA	bulk/OF
TZN@EuB4C			X	TZN	bulk/OF

Figure 1 displays images of several samples produced in this study. In panel 1­(a), a photograph of TZN@[Eu­(BTC)­(PDC)] demonstrates a uniform coating layer on one face of the glass surface, while the other faces were polished to remove the coating. Panel 1­(b) showcases the PNKA@EuBTC (transparent glass) and TZN@EuBTC (yellowish glass) samples under white light, whereas panel 1­(c) displays their intense luminescence under UV light centered at 254 nm. Furthermore, panel 1­(d) presents a cross-sectional image obtained via optical microscopy, allowing the measurement of the coating thickness, which averages 20 μm, and panel 1­(e) shows the same sample irradiated with blue LED light centered at 405 nm. Finally, Figure 1f presents optical microscopy images of TZN@EuBTC and PNKA@EuBTC samples, revealing distinct differences in film appearance and adhesion despite identical synthesis conditions.

**Figure 1:**
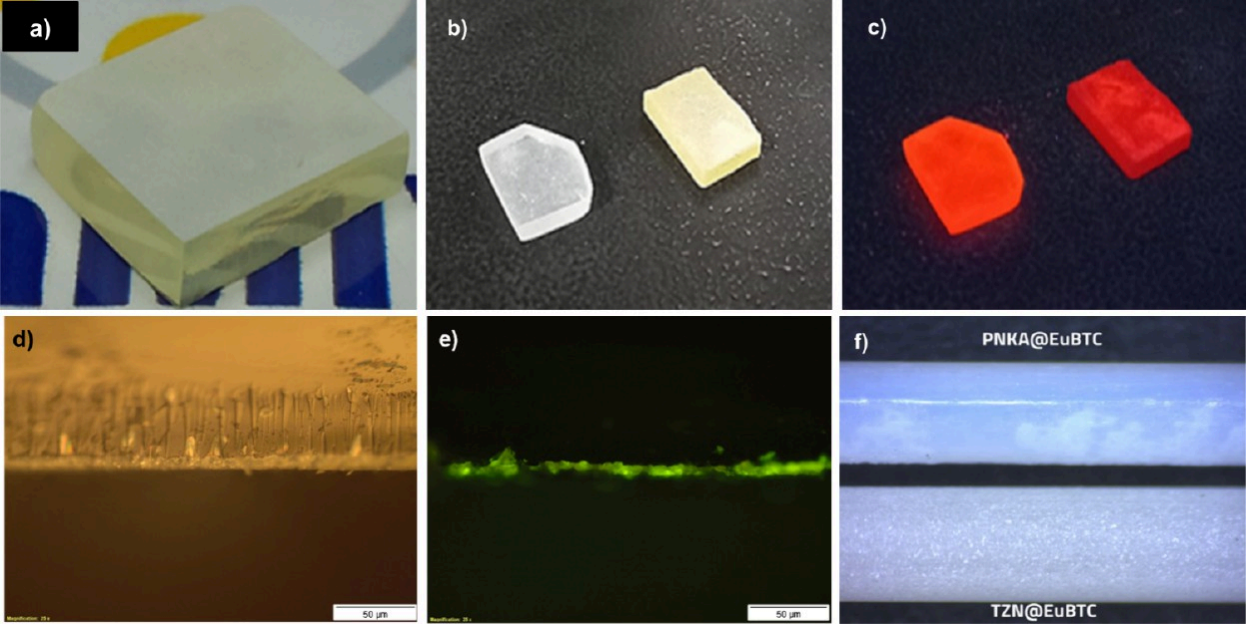
Photographs of (a) TZN@[Eu­(BTC)­(PDC)]; (b) PNKA@EuBTC and TZN@EuBTC under thin light and (c) irradiated by UV-light at 254 nm; optical microscopy images of TZN@EuBTC under (d) white light and (e) blue light (405 nm); and (f) TZN@EuBTC and PNKA@EuBTC fibers, showing distinct coating appearances.

**Figure 2:**
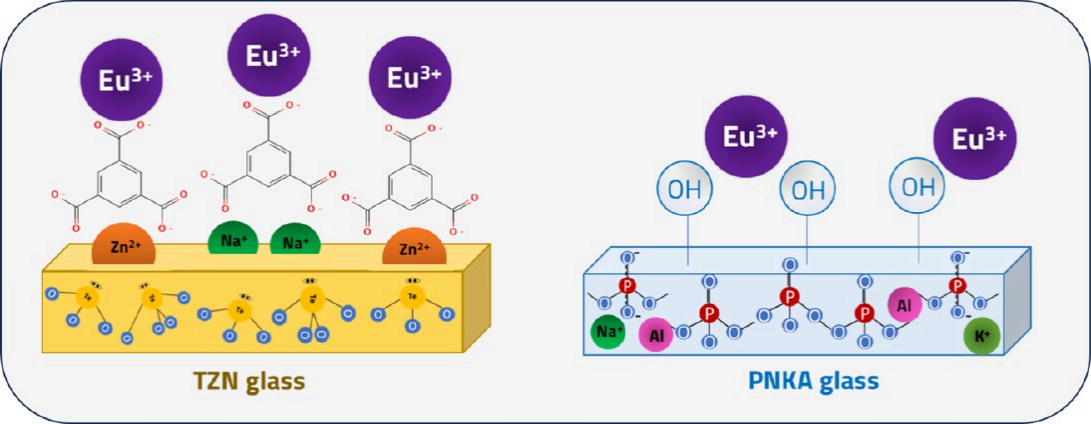
Representative scheme for the interaction between the glass systems and Ln-CP components.

To investigate the interaction between the glass and the CP, synthesis procedures were conducted using individual CP components, specifically, either the carboxylate ligand, BTC, or the Eu^3+^ precursor. Adhesion tests were performed using OFs as the glass substrate. TZN and PNKA substrates were exposed to the BTC ligand in an aqueous H_3_BTC solution at a pH of 3.6. Figure S1a illustrates the BTC ligand coverage on the TZN fiber, resulting in a crystalline coating, while the PNKA fiber exhibited no adhesion of this species. However, exposure to EuCl_3_ solution resulted in a nonuniform coating on the PNKA surface, whereas no adhesion was observed on the TZN fiber (Figure S1b). This behavior suggests an interaction between the carboxyl groups and the TZN glass, as well as potential coordination of Eu^3+^ with the negatively charged groups on the PNKA surface, influenced by the glass composition and structural properties.

As previously reported in the literature, aluminum-phosphate glasses exhibit good transparency in the UV–visible range but limited infrared transmission due to the high concentration of hydroxyl (OH) groups within their matrix.^38^ Consequently, a significant amount of OH groups is expected on the glass surfaces, which can interact with cations such as Eu^3+^. In contrast, tellurite glasses are commonly used in mid-infrared applications, showcasing relatively low OH concentrations alongside high network connectivity attributed to the formation of TeO_4_ units. These intrinsic characteristics play an important role in understanding their interaction with carboxylate ligands.

Given the acidic medium during the synthesis of Ln-CPs, it is plausible that a chemical attack on the glass surface occurs, leading to the removal of surface hydroxyl groups.^39^ This process could expose cationic species, such as Zn^2+^, embedded within the glass matrix, facilitating their interaction with carboxylate ligands. These species may act as coordination sites for the carboxylate groups of the ligands, as illustrated in Figure 2. This phenomenon may explain the formation of coatings on other tellurite glass matrices, such as TZNF15 and TN, whereas the PNKA matrix may exhibit a greater affinity for Eu^3+^ due to its higher OH content.

To elucidate the structures of the synthesized coatings, XRD analyses were performed on the deposited films (on glass substrates) and on the corresponding powders collected from the reactor (separated by centrifugation and dried at 80 °C for 5 h). As shown in Figure S2, the diffractograms reveal that the glass composition significantly influences the formation of the CP structure. TZN@[Eu­(BTC)­(PDC)] exhibits a greater number of diffraction peaks matching the powder sample and displays sharper peaks compared to TZNF15@[Eu­(BTC)­(PDC)], indicating a higher degree of crystallinity. Moreover, TZN@EuBTC shows broader peaks, suggesting partial disorder or the presence of amorphous regions, whereas TZN@EuB4C presents more pronounced crystalline features with narrow, intense peaks. These patterns were derived from thin-film coatings grown *in situ* on substrates and not from conventional powders. Such growth conditions^40^ may introduce factors like preferred orientation or substrate-induced effects on crystal growth. The literature confirms the EuBTC structure typically crystallizes in a monoclinic system (Cc or C2/m), featuring Eu^3+^ in a tricapped trigonal prismatic geometry, forming ribbon-like motifs stabilized by hydrogen bonding and π–π interactions.^41,42^ In contrast, EuB4C frameworks form a three-dimensional network using EuO_9_ polyhedral and pyromellitic acid ligands, which generate 1D chains that assemble into layered, interconnected 3D networks via oxocarboxylate bridges.^36,43^

The morphology of Ln-CPs coated on TZN glasses was analyzed using FEG-SEM combined with EDX. As depicted in Figure S3, the TZN@Eu­(BTC)­(PDC) sample exhibits the formation of rod-like crystals, typical for carboxylate CPs.^44^ Moreover, EDX analysis maps reveal a predominant elemental distribution of C, Eu, and O across its surface, indicating an extensive coverage by the Ln-CP. The TZN@Eu­(BTC)­(PDC) sample was also synthesized under identical conditions, with the reaction time shortened to 10 min. The images obtained at the shorter reaction time (Figure 3) reveal that the morphologies of the Ln-CPs are surprisingly different from those shown in Figure S3. The sample in Figure 3 exhibits structures with a symmetric coralloid pattern,^45^ and the EDX mapping confirms that these structures are composed of CP elements (C, O, and Eu). This coralloid shape is attributed to rapid nucleation process and is characteristic of MOFs containing BTC ligands.^46,47^ Although the structures are similar to those of MOFs, they are classified here as CPs because the *in situ* synthesis prevents porosimetry measurements required to confirm their porous nature. Another observation from Figure 3 is the presence of cubic structures, indicating fragments of glass detached from the bulk. This reinforces the possibility of chemical attacks due to the acidic pH of the reaction medium, which may also influence the adhesion of Ln-CP to the substrate.

**Figure 3:**
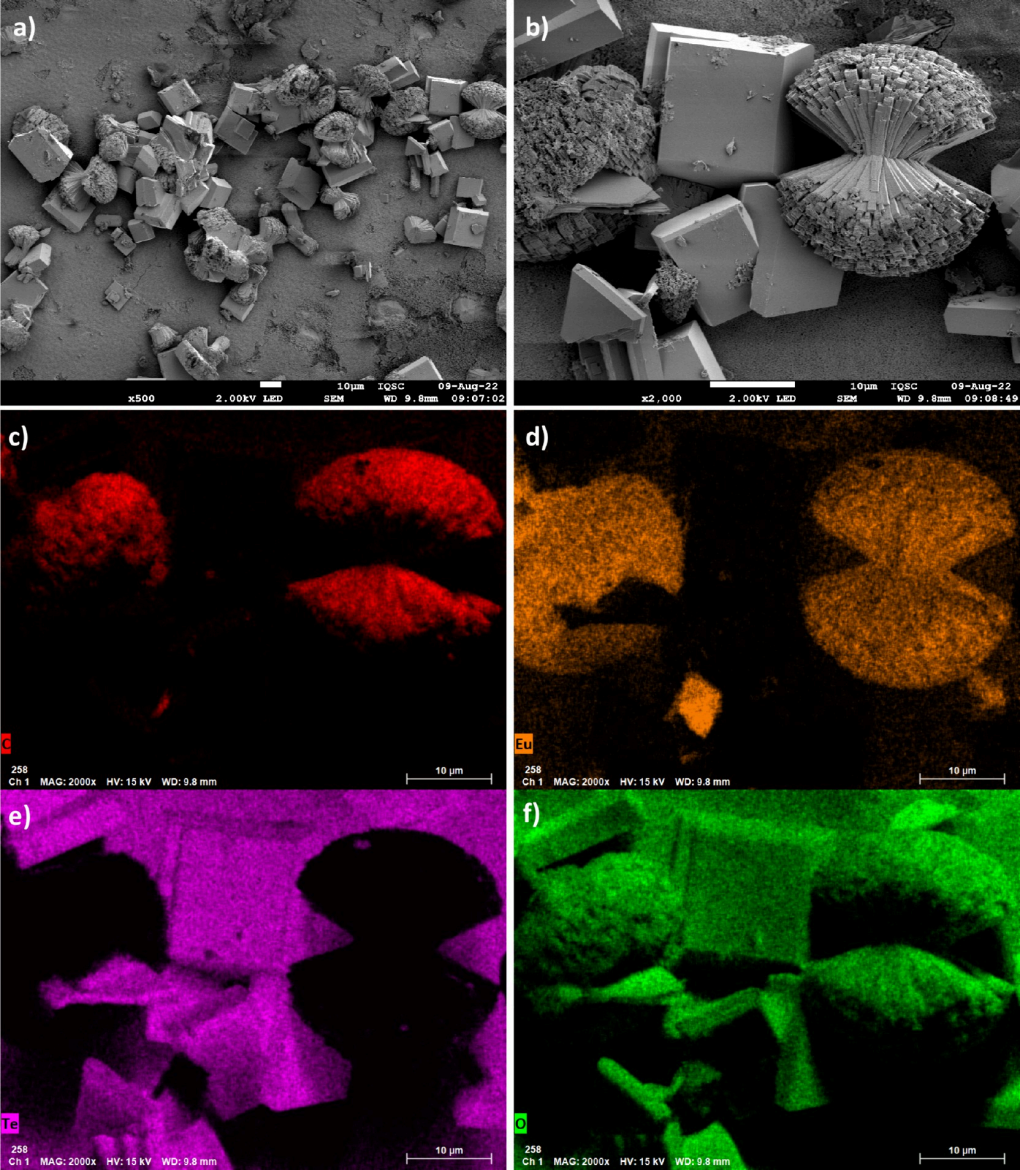
SEM-FEG images of the surface of TZN@[Eu­(BTC)­(PDC)]: (a, b) with different magnifications; and EDX analysis with the mapping of the elements: (c) C (red), (d) Eu (yellow), (e) Te (purple), and (f) O (green).

Other compositions were morphologically characterized by using FEG-SEM. Figure 4 displays the surface images of the TZN@EuBTC, TN@EuBTC, and TZN@EuB4C samples. A comparison of Figure 4a,b reveals similar surface morphologies between the TZN@EuBTC and TN@EuBTC samples. However, the TZN@EuBTC sample exhibits a greater amount of deposited material on its surface, suggesting stronger attraction between the BTC ligands and the TZN glass, likely facilitated by the presence of Zn^2+^. Figure 4c,d shows the surface morphology of the TZN@EuB4C sample at different magnifications, revealing the presence of spherulitic structures.

**Figure 4:**
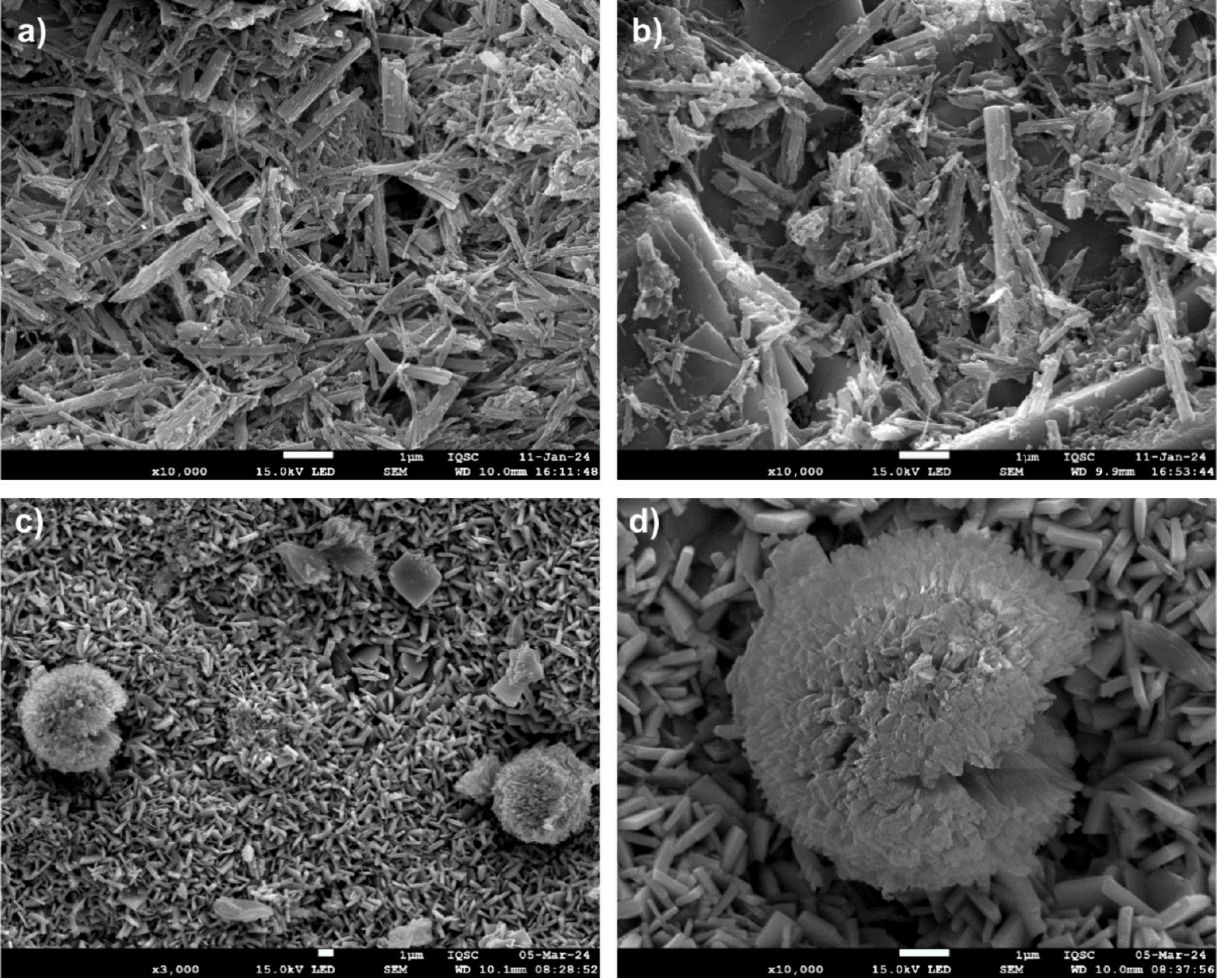
SEM-FEG images of the surfaces of (a) TZN@EuBTC at 10,000× magnification; (b) TN@EuBTC at 10,000× magnification; (c) TZN@EuB4C at 3000× magnification; and (d) TZN@EuB4C at 10,000× magnification.

### 3.2 PL Characterization

The PL behavior of glass@Ln-CPs samples was also investigated. First, the solid-state PL spectra of TZNF15@[Eu­(BTC)­(PDC)] are shown in Figure[Fig fig5]. The excitation spectrum, monitored at 617 nm, reveals a broad absorption band centered at 301 nm, attributed to intraligand (IL) transitions, along with relatively narrow bands in the 350–550 nm region, assigned to f–f transitions. The most intense band in this region corresponds to the ^7^F_0_ → ^5^L_6_ transition centered at 394 nm. The emission spectra of TZNF15@[Eu­(BTC)­(PDC)] were recorded under excitation at 301 (red line, ligand excitation) and 394 nm (blue line, Eu^3+^ excitation), as also shown in Figure 5. When excited at 301 nm, the composite exhibits a broad band with a maximum at 420 nm, attributed to IL transitions, along with three bands corresponding to Eu^3+^ transitions, centered at 598 nm (^5^D_0_ → ^7^F_1_), 617 nm (^5^D_0_ → ^7^F_2_), and 700 nm (^5^D_0_ → ^7^F_4_). This indicates the occurrence of the antenna effect by energy transfer from the ligand to the metal center.^48^ Furthermore, the PL spectrum obtained with excitation at 394 nm shows, in addition to the previously observed characteristic Eu^3+^ emissions, two additional weak f–f transitions at 580 nm (^5^D_0_ → ^7^F_0_) and 653 nm (^5^D_0_ → ^7^F_3_). In both excitation scenarios, the most intense transition is the ^5^D_0_ → ^7^F_2_ transition, which dominates due to the low-symmetry environment of the Eu^3+^ centers.^49^ The presence of the ^5^D_0_ → ^7^F_0_ emission, a symmetry-forbidden transition, further suggests a noncentrosymmetric site around Eu^3+^.^50^ Additionally, the decay time of the emission at 617 nm (inset of Figure 5) was fitted to a double-exponential function ((τ_1_ = 0.366 ms (89.62%); τ_2_ = 1.22 ms (10.38%)), indicating the existence of different chemical microenvironments around the Eu^3+^ centers in the sample. A preexponential weighted lifetime (τ_M_ = Στ_i_B_i_/ΣB_i_) was calculated to allow comparison with lifetime values obtained from single exponential fitted curves.^51^

**Figure 5:**
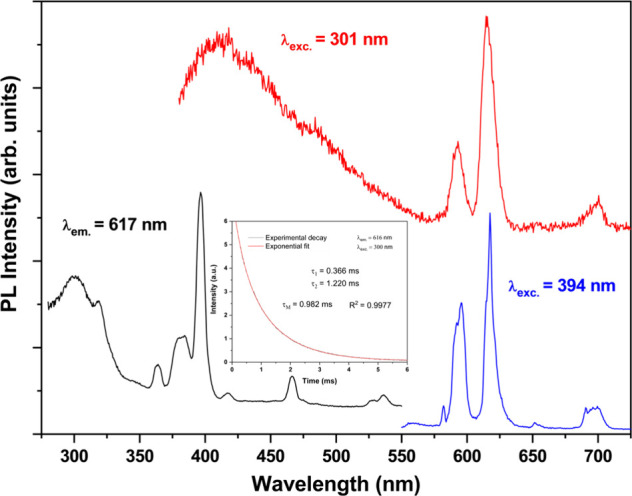
PL spectra of TZNF15@[Eu­(BTC)­(PDC)]; the emission at 617 nm was monitored in the excitation spectra (black), and two emission spectra were recorded under excitation at 394 nm (blue) and 301 nm (red). Inset: experimental luminescence decay and the corresponding biexponential fit curve to calculate the decay time for this sample.

The TZN@EuBTC and TZN@EuB4C samples were also analyzed in terms of their PL properties. Figure 6a depicts the excitation spectra of both samples with the emission monitored at 616 nm. In addition to the characteristic Eu^3+^ f–f transitions, a broad ligand absorption band is observed around 300 nm, with the maxima at 295 nm for TZN@EuBTC and 310 nm for TZN@EuB4C. Despite this slight difference in the excitation maxima, it is noteworthy that the ligand excitation band is significantly more intense for the EuB4C sample, compared to the excitation intensity of the Eu^3+^ ^7^F_0_ → ^5^L_6_ transition centered at 395 nm. This indicates a stronger contribution of the ligand to the energy-transfer process associated with the monitored emission, suggesting a more effective antenna effect in EuB4C composition. This observation is consistent with reports in the literature, which show that despite similar S_0_ → S_1_ transition energies for both ligands, CPs containing H_4_B4C typically exhibit a lower triplet-state (T_1_) energy compared to those based on H_3_BTC.^52–55^ A comparative energy-level diagram, constructed using average T_1_ values from previous reports, is presented in Figure 6b. Specifically, the energy gap between the ^5^D_0_ level of Eu^3+^ and the ligand T_1_ state is estimated to be ≈7500 cm^–1^ for EuBTC, while this difference is significantly reduced to ≈3500 cm^–1^ for EuB4C. This reduced energy gap directly corroborates the higher efficiency of the energy-transfer process observed in EuB4C.^56,57^

**Figure 6:**
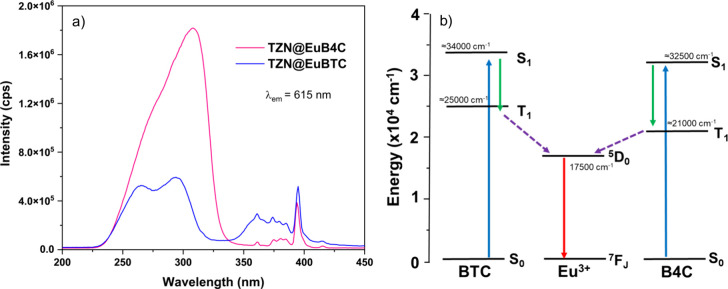
(a) Excitation spectra of TZN@EuBTC and TZN@EuB4C samples monitored at 616 nm; and (b) comparative energy-level diagram illustrating the ligand-centered singlet (S_1_) and triplet (T_1_) states and the ^5^D_0_ excited level of Eu^3+^ for EuBTC and EuB4C CPs.

The emission spectra for TZN@EuBTC and TZN@EuB4C are shown in Figure 7. Both samples were excited at three different wavelengths: 310 nm (ligand excitation), 360 nm (an intermediate region between the ligand and Eu^3+^ excitation), and 395 nm (direct Eu^3+^ excitation). For the TZN@EuBTC sample (Figure 7a), the emission intensity is similar when excited at both 310 and 395 nm. However, for the TZN@EuB4C sample (Figure 7b), a significant enhancement in emission is observed with excitation at 310 nm, showing an intensity approximately five times higher than that achieved with direct Eu^3+^ excitation at 395 nm. This considerable increase highlights the efficiency of the antenna effect in this composition, where the Eu^3+^ luminescence, typically originating from forbidden f–f transitions, is significantly amplified when the CP ligand is excited and this energy is subsequently transferred to the Eu^3+^. The insets of Figure 7a,b display the experimental decay curves and their exponential fits. The decay time for TZN@EuBTC was calculated as 0.201 ms using the pre-exponential weighted lifetime method,^51^ whereas the TZN@EuB4C sample exhibited a single-exponential fit with a decay time of 0.298 ms. These results reinforce the conclusion that the antenna effect is more efficient in the TZN@EuB4C sample, as longer decay times are typically associated with enhanced energy-transfer processes.^58^

**Figure 7:**
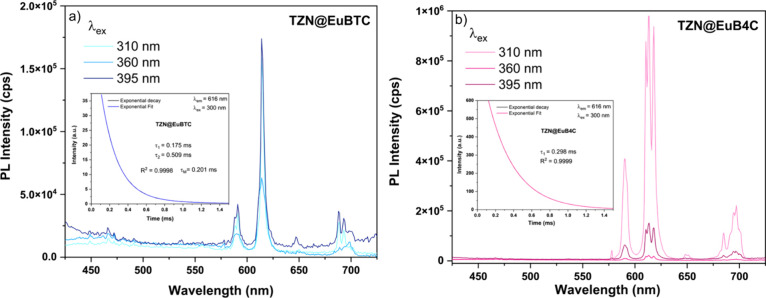
(a) Emission spectra of TZN@EuBTC excited at 310, 360, and 395 nm. (b) Emission spectra of TZN@EuB4C under the same excitation wavelengths. Insets: Experimental decay curves (λ_ex_ = 300 nm; λ_em_ = 616 nm) and their corresponding exponential fits, with decay times of 0.201 ms for TZN@EuBTC and 0.298 ms for TZN@EuB4C.

### 3.3 Luminescence Sensing of Small Organic Molecules

Preliminary sensing tests for several organic compounds were performed by using a spectrofluorometer. A sealed quartz cuvette with a plastic lid was employed to minimize the analyte evaporation. The sample was positioned diagonally within the cuvette to ensure optimal exposure and measurement accuracy. The TZN@EuBTC sample was immersed in each of the organic solvents and excited at 300 nm, and the luminescence spectra were collected (Figure 8). In these conditions, a complete quenching of the Eu^3+^ luminescence was observed in acetone, which could be attributed to the intrinsic absorption of acetone in this spectral range.^59^

**Figure 8:**
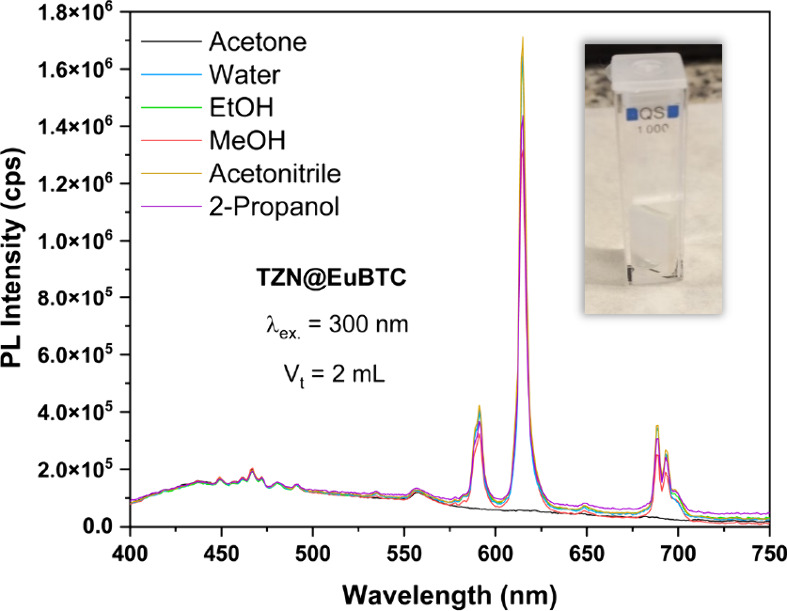
Luminescence spectra of TZN@EuBTC when immersed in organic solvents and excited at 300 nm. Inset*:* Picture of the sealed quartz cuvette containing the TZN@EuBTC sample.

To further examine the effect of acetone on the luminescence of TZN@EuBTC, the sample was immersed in aqueous solutions containing varying concentrations (volume %) of the analyte and its PL spectrum was recorded under 310 nm excitation. Figure 9a shows an exponential luminescence quenching with an increasing acetone concentration. Moreover, when a lower concentration range is used, a more linear decrease in luminescence intensity is observed (Figure S4), indicating a direct correlation between the luminescence intensity and the analyte concentration. However, as mentioned above, this sensing mechanism is likely due to the acetone absorption at 310 nm, which reduces the effective intensity of the excitation light reaching the CP. Acetone absorption was analyzed across a concentration range of 0–1.75% by volume, and it can be seen in Figure S5. The absorbance at 310 nm exhibits a linear increase at concentrations above 0.40% by volume, clearly demonstrating that the inner filter effect significantly influences the changes in luminescence intensity.^60^ In contrast to the behavior observed for TZN@EuBTC, the TZN@EuB4C sample demonstrated a luminescence enhancement when exposed to low acetone concentrations (up to 1.0%, v/v), as shown in Figure 9b. This enhancement suggests that acetone is adsorbed within the CP pores, facilitating direct energy transfer to the Eu^3+^ and thereby increasing luminescence through a guest-induced phenomenon.^61,62^ At higher concentrations (Figure 9c), the Eu^3+^ emission intensity begins to decrease but remains higher than that in the initial spectra obtained with the sample immersed in water. This behavior indicates that both the luminescence enhancement due to energy transfer from adsorbed acetone molecules and the quenching, caused by the inner filter effect from acetone in solution, occur simultaneously at high acetone concentrations. The procedure was conducted three times using the same sample. The reusability of the composite was verified by measuring the initial and maximum luminescence in each series, as shown in Figure 9d.

**Figure 9:**
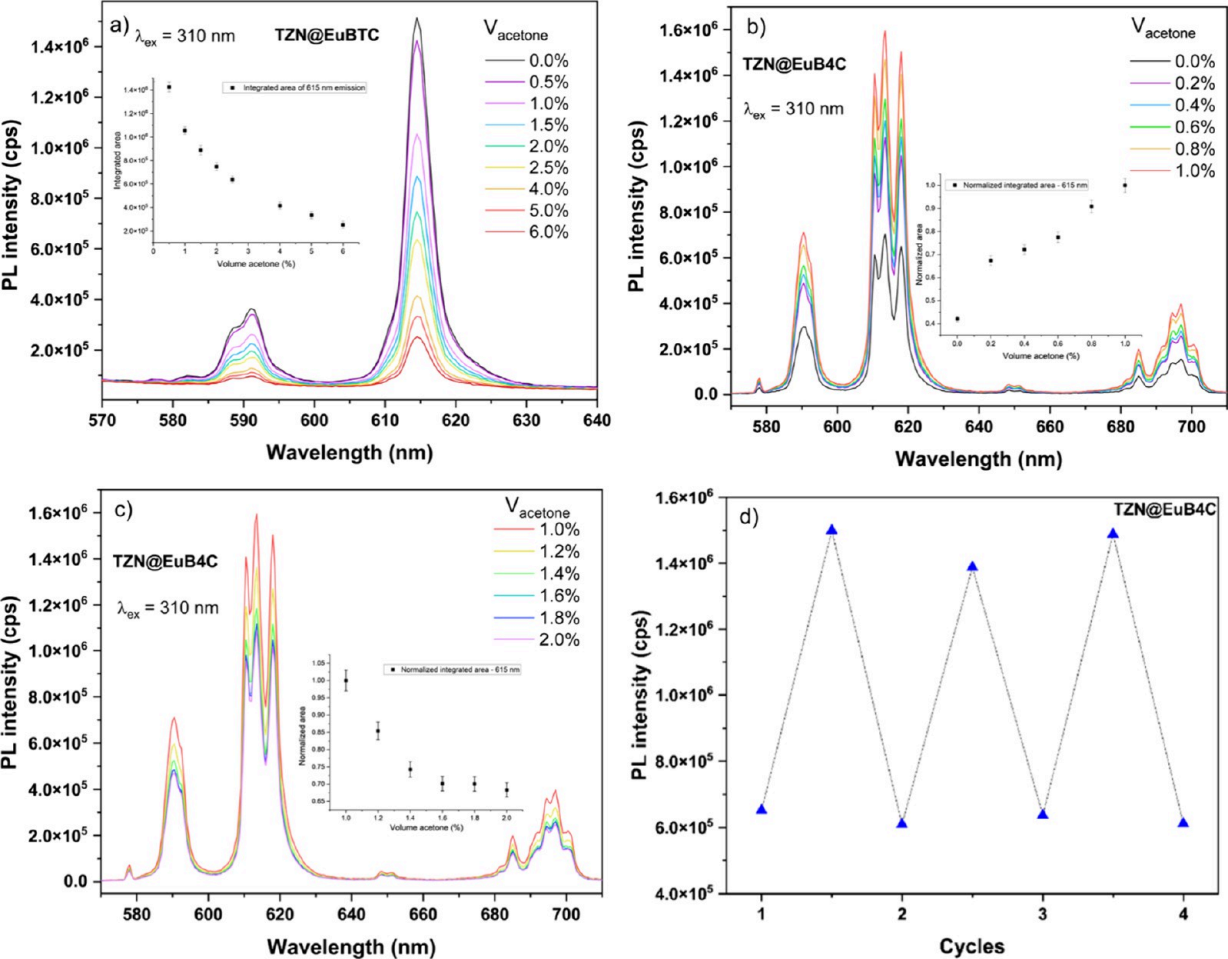
(a) Luminescence in the TZN@EuBTC sample with increasing volumes of acetone (up to 6.0%) under 310 nm excitation; (b) enhanced luminescence response of TZN@EuB4C to low concentrations (up to 1.0% v/v) of acetone in water; (c) decrease of the Eu^3+^ emission intensity at higher concentrations, both excited at 310 nm; and (d) initial and maxima PL intensities of TZN@EuB4C exposed to acetone in water.

To rationalize the sensing mechanism of acetone by composite TZN@EuB4C, a proposed model is illustrated in Figure 10a. As mentioned above, when acetone molecules are adsorbed onto the CP layer, they can efficiently absorb excitation light (Figure 10b). Due to the proximity of the S_1_ states of the acetone and the carboxylate ligand (see the energy diagram in Figure 10c), the analyte can assist the Ln-CP excitation process.^63^ This suggests that the antenna effect is more efficient under these conditions, leading to an increase in the Eu^3+^ emission intensity. After the CP pores become saturated, an excess of acetone remains in solution, resulting in an increased absorption of excitation light, similar to the inner filter effect discussed for the TZN@EuBTC. Consequently, acetone sensing by TZN@EuB4C shows a dynamic interplay between the turn-on and inner filter effects. Initially, this competition results in an enhancement of the luminescence intensity; however, once analyte adsorption is saturated, further luminescence quenching occurs, driven by the absorption of the excitation light by free acetone molecules in the solution.

**Figure 10:**
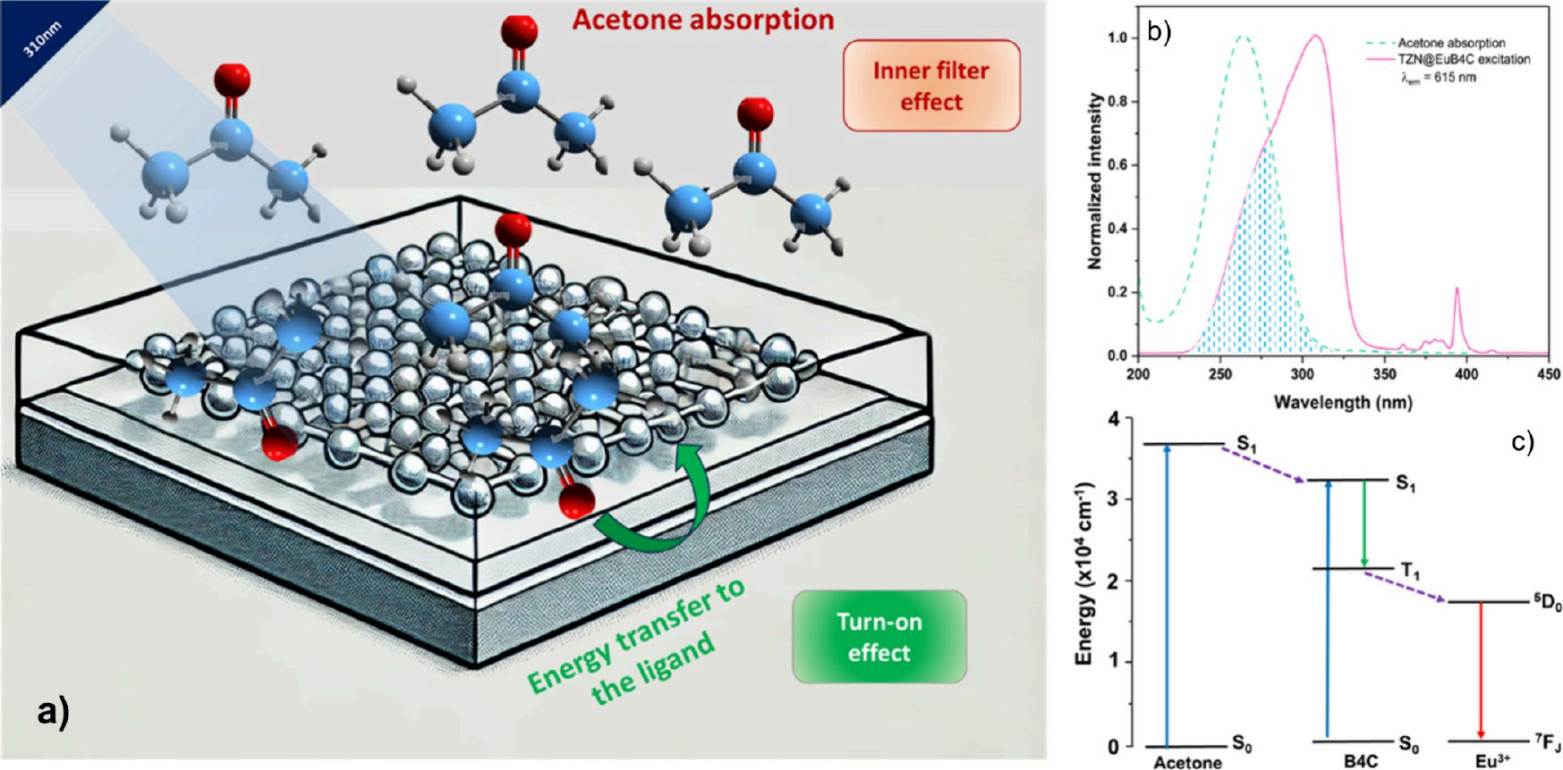
Proposed sensing mechanism for acetone in the TZN@EuB4C sample: (a) scheme illustrating the interaction between acetone molecules both adsorbed on the CP layer and in solutions, highlighting the competition between the turn-on and inner filter effects; (b) comparison between the acetone adsorption and TZN@EuB4C excitation spectra, with the intersectional area highlighted; and (c) energy-level diagram showing the proximity of the S_1_ states of the B4C ligand and acetone, facilitating efficient excitation energy transfer and enhancing the Eu^3+^ emission intensity.

Following the procedure applied with acetone, small amounts of 2-pentanone in water were also tested for detection, as shown in Figure S6. At low concentrations of 2-pentanone, an increase in luminescence was observed, with the emission intensity peaking at 0.4% by volume, followed by a decline at higher concentrations. This behavior, consistent with that observed for acetone, indicates the involvement of similar underlying mechanisms. The luminescence response demonstrates excellent selectivity for detecting ketones, even at low concentrations, underscoring its potential since several ketones function as biomarkers associated with cancer and other diseases.^64–67^ In addition, acetylacetone, a diketone, was analyzed for its luminescence response, as shown in Figure S7. A strong quenching effect was observed at low concentrations, specifically below 0.15% by volume. Replicate tests confirmed that this quenching was irreversible, likely due to the coordination of acetylacetone with Eu^3+^, leading to modification of the CP structure. This irreversible structural alteration compromises the material’s suitability for sensing diketones, as the changes prevent a reliable, reversible luminescent response.

### 3.4 Luminescence Sensing Using Coated OFs

The attenuation curves of single-index TZN OFs (170 μm in diameter) fabricated using different crucible materials exhibit relatively low optical losses in the 550–700 nm range, making them suitable for guiding Eu^3+^ emission (Figure S8). The luminescence of the coating was measured using a 15 cm long OF with varying diameters to assess the feasibility of monitoring the luminescence (Figure S9). The fiber tip was illuminated by using a UV lamp emitting at 254 nm, while the emitted light was collected at the opposite end through a commercial ZrF_4_ (ZBLAN) fiber coupled to a portable spectrometer. The results revealed the characteristic emissions of Eu^3+^, with the most intense one corresponding to the ^5^D_0_ →^7^F_2_ transition centered at 612 nm. An improvement of the signal intensity was observed for fibers with larger diameters (from 270 μm to 1 mm). The micrograph of the TZN@EuB4C fiber (Figure S10a) reveals a visually homogeneous coating. Furthermore, the detailed SEM image (Figure S10b) confirms the good adhesion of the CP to the fiber surface. The coating thickness was determined by optical microscopy (using a 100x objective) and ranged from 2.7 to 9.6 μm, a variation directly dependent on the synthesis time (Figure S10c–f).

To perform sensing measurements using 1 mm OFs coated with Ln-CP, we established an experimental setup that enables the immersion of the fiber’s tip into the analyte solution while connecting it to a spectrometer for luminescent signal collection (see Figure S11). Subsequently, the TZN@EuB4C fiber sample was immersed in a cuvette filled with water and connected to a vertically positioned fiber optic spectrometer. Illumination was performed from outside the cuvette. Small volumes of acetone were added to the cuvette starting at 0.05% for the initial measurements. Figure 11a–c illustrates the luminescence intensity response as the acetone concentration increases, revealing a maximum emission at a concentration of 1.0% by volume. At higher concentrations, a decrease in emission intensity is observed, which can be attributed to the saturation of analyte adsorption and its increased presence in solutions, leading to greater absorption of the excitation light. Figure 11d shows the linear dependence of emission intensity at 616 nm with the acetone concentration within the range of 0.05–0.30% by volume, showing a slope of 0.25 ± 0.01. The minimum acetone concentration tested (0.05%, or 500 ppm) is higher than the typical detection limits (20–100 ppm) reported for powder-based CP systems, which are generally investigated as suspensions in solution. This discrepancy likely results from two factors: the smaller analyte interaction area inherent to our solid-state composite films and specific experimental constraints. In our methodology, small volumes of acetone were introduced directly into the cuvette without removing it from the fluorimeter, a step taken to ensure the same coating region was monitored and to minimize intensity fluctuations. Despite this limitation, the reproducible and reversible luminescence response highlights the significant potential of this system for optical sensing applications.

**Figure 11:**
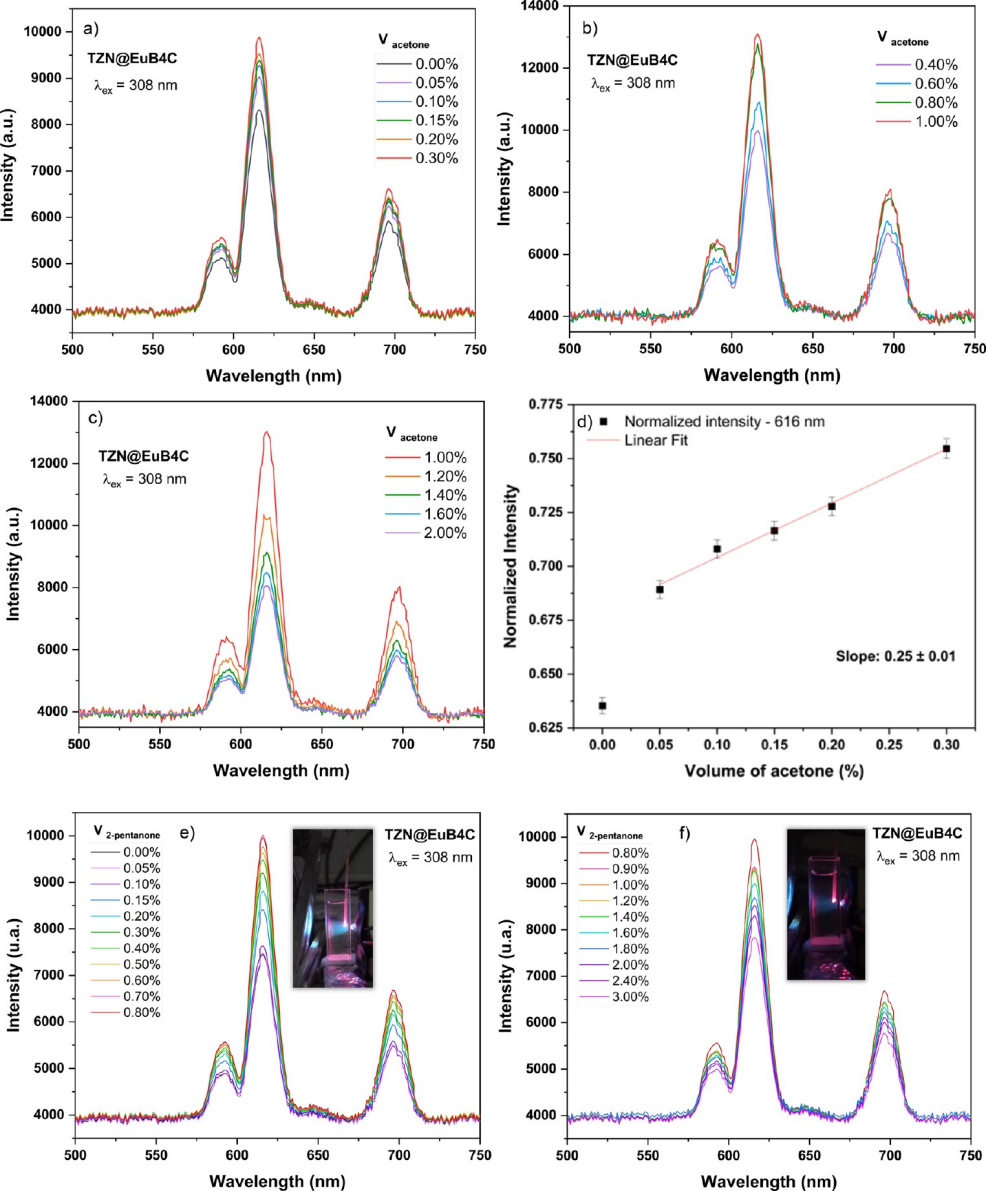
Luminescence intensity of the TZN@EuB4C fiber with varying acetone concentrations, in different ranges: (a) from 0.00 to 0.30%; (b) from 0.40 to 1.0%; and (c) from 1.0 to 2.0% (v/v); (d) linear dependence between the emission intensity at 616 nm and the acetone concentration in the 0.05–0.30% (v/v) range; luminescence response of the TZN@EuB4C fiber to 2-pentanone: (e) enhancement of emission intensity with varying concentrations of 2-pentanone, with the maximum intensity at 0.70%; and (f) quenching of luminescence at concentrations above 0.80%.

The same composition was tested for the detection of 2-pentanone, and a similar enhancement in the Eu^3+^ emission intensity was observed (Figure 11e), consistent with the behavior reported in previous tests. In this case, the maximum intensity was recorded at 0.70%, slightly higher than the values obtained for the bulk samples. Despite this difference and the nonlinear response, a clear trend of increasing emission intensity with the gradual addition of the analyte was observed up to this concentration. At concentrations above 0.80% (Figure 11f), luminescence quenching occurred, likely due to the higher concentration of free analyte in solution and its intrinsic absorption in the UV region. Notably, the emission intensity remained nearly constant between 0.90 and 1.40%, which may result from the opposing effects of quenching and enhancement associated with analyte absorption and energy transfer to the emitting center. Although the response is not strictly linear, these results confirm that the developed material exhibits sensitivity and an optical response to the presence of carbonyl compounds, particularly in the ketones tested (acetone and 2-pentanone).

## 4 Conclusions

This work reports a rapid synthesis of new and stable glass@Ln-CP and coated optical-fiber@Ln-CP composites. Luminescent Ln-CP coatings combining Eu^3+^ and carboxylate ligands were successfully formed and demonstrated good adhesion to tellurite glass substrates. The coated OFs represent a promising material due to their ability to guide optical signals over long distances, enabling the development of remote sensors for diverse analytes. The findings underscore the potential of these materials for chemical sensing, especially for the detection of carbonyl compounds. The successful integration of Ln-CPs at the tip of OFs, especially TZN-based fibers, demonstrates the feasibility of using these composites in real-time sensing. The enhanced luminescence response observed at low ketone concentrations (<0.5% v/v) indicates their high selectivity, while the linear response within specific concentration ranges confirms their reliability for quantitative detection. These features position the developed materials as promising candidates for challenging demands, such as industrial monitoring, environmental analysis, and particularly for the detection of biomarkers in health monitoring and disease diagnostics. This research also provides opportunities for further optimization, including enhancing the adhesion of CPs to fiber surfaces and exploring alternative fiber materials to improve sensor performance. Overall, the outcomes provide valuable insights into the development of optical sensors based on glass@Ln-CPs composites, combining the luminescent properties of metal–organic materials with the light-guiding ability of OFs.

## Supplementary Material



## Notes

Additional experimental details and results, including photographs of samples and measurement setup, SEM and optical microscopy images, XRD diffractograms, PL and absorption spectra, and attenuation curves of single-index OFs (PDF)
