# Supplementary material for: In Situ Growth of Lanthanide Coordination Polymers on Oxide Glass and Optical Fibers: A Promising Material for Chemical Sensing
Source: ACS Appl Mater Interfaces. 2025 Dec 17;17(52):70667–79. doi: 10.1021/acsami.5c16933 (PMC12766679; doi:10.1021/acsami.5c16933)
Supplement: Supplementary file 1 [file am5c16933_si_001.pdf]

Supporting Information

In situ growth of lanthanide coordination polymers on oxide glass and optical fibers: a promising material for chemical sensing

Renato G. Capelo[1,2*], Francis D. R. Garcia[1], Clément Strutynski[2], Frédéric Désévédavy[2], Gregory Gadret[2], Caroline M. da Silva[3], Guilherme Arroyos[3], Regina C. G. Frem[3], Guillermo Orellana[4], Frédéric Smektala[2], Danilo Manzani[1*]

[1] São Carlos Institute of Chemistry, University of São Paulo, 13566-590, São Carlos, SP, Brazil.

[2]Laboratoire Interdisciplinaire Carnot de Bourgogne ICB UMR 6303, Université Bourgogne Europe, CNRS, 21000, Dijon, France.

[3]Institute of Chemistry, São Paulo State University, 14800-900, Araraquara, SP, Brazil.

[4]Facultad de Ciencias Químicas, Universidad Complutense de Madrid, 28040, Madrid, Spain.

*Corresponding authors:

Danilo Manzani
Instituto de Quimica de São Carlos (IQSC)
Universidade de São Paulo
São Carlos – SP – Brazil
E-mail: dmanzani@usp.br

Renato Grigolon Capelo
Instituto de Quimica de São Carlos (IQSC)
Universidade de São Paulo
São Carlos – SP – Brazil
E-mail: renatocapelo@usp.br

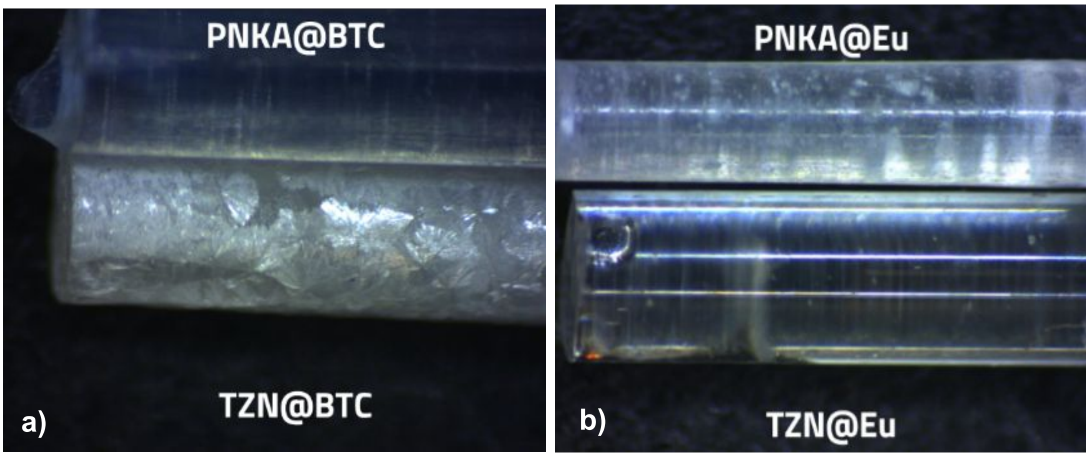

Figure S1. Optical microscopy images of glass substrates after exposure to individual CP components: (a) BTC ligand coating on TZN fiber with crystalline aspects and no adhesion on PNKA fiber; (b) $EuCl_3$ coating on PNKA fiber with non-uniform coverage and no adhesion on TZN fiber.

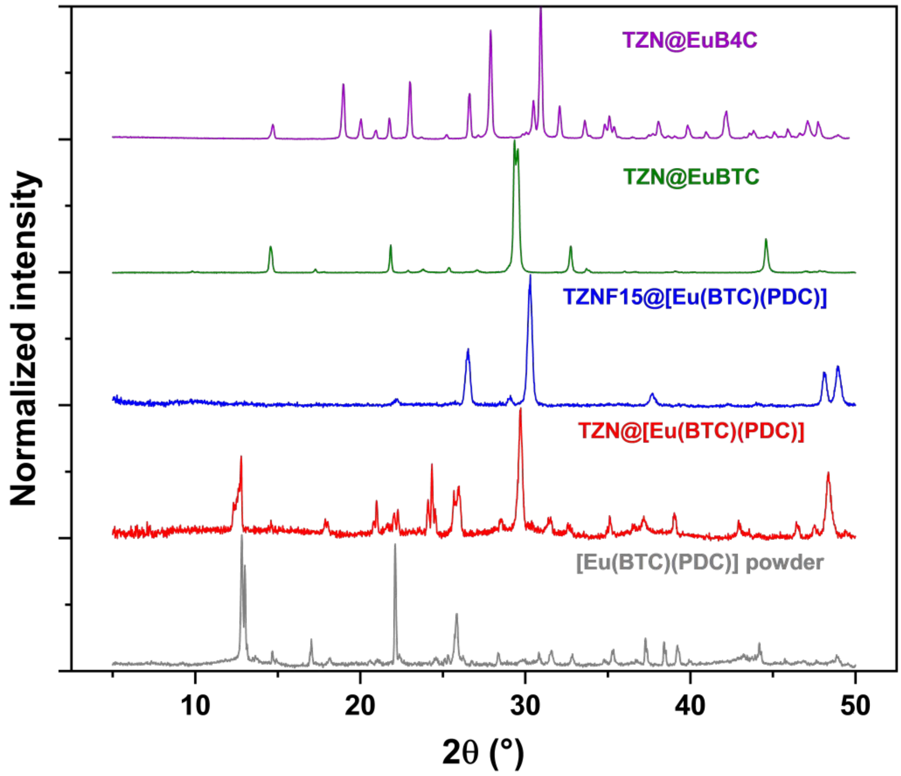

Figure S2. Diffractograms of synthesized Ln-CPs with measurements of precipitates obtained after the syntheses (in powder form) and coatings on the glass bulk samples: TZN@[Eu(BTC)(PDC)], TZNF15@[Eu(BTC)(PDC)], TZN@EuBTC and TZN@EuB4C.

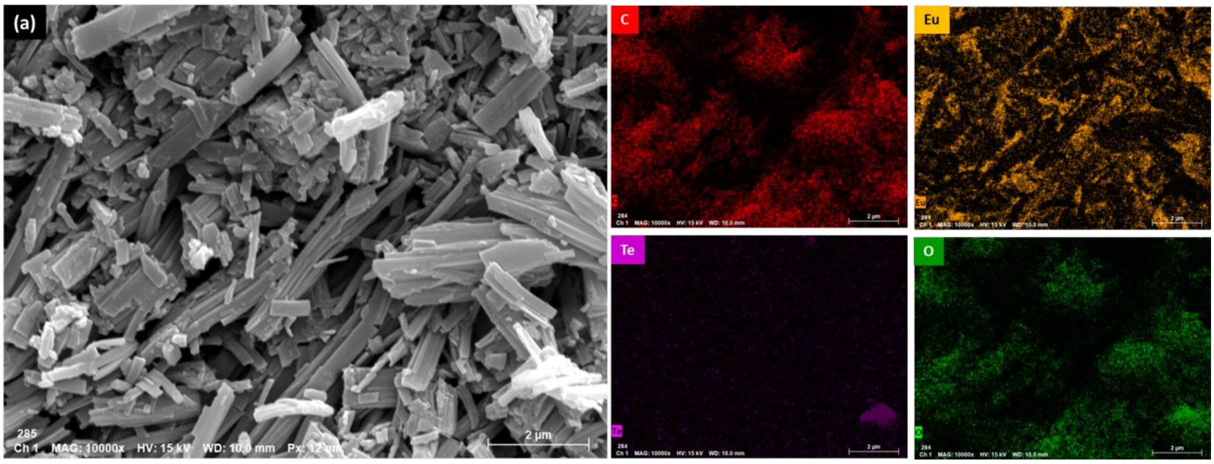

Figure S3. FEG-SEM images and EDX analysis of (a) TZN@[Eu(BTC)(PDC)] with the mapping of the elements C (red), Eu (yellow), Te (purple), and O (green).

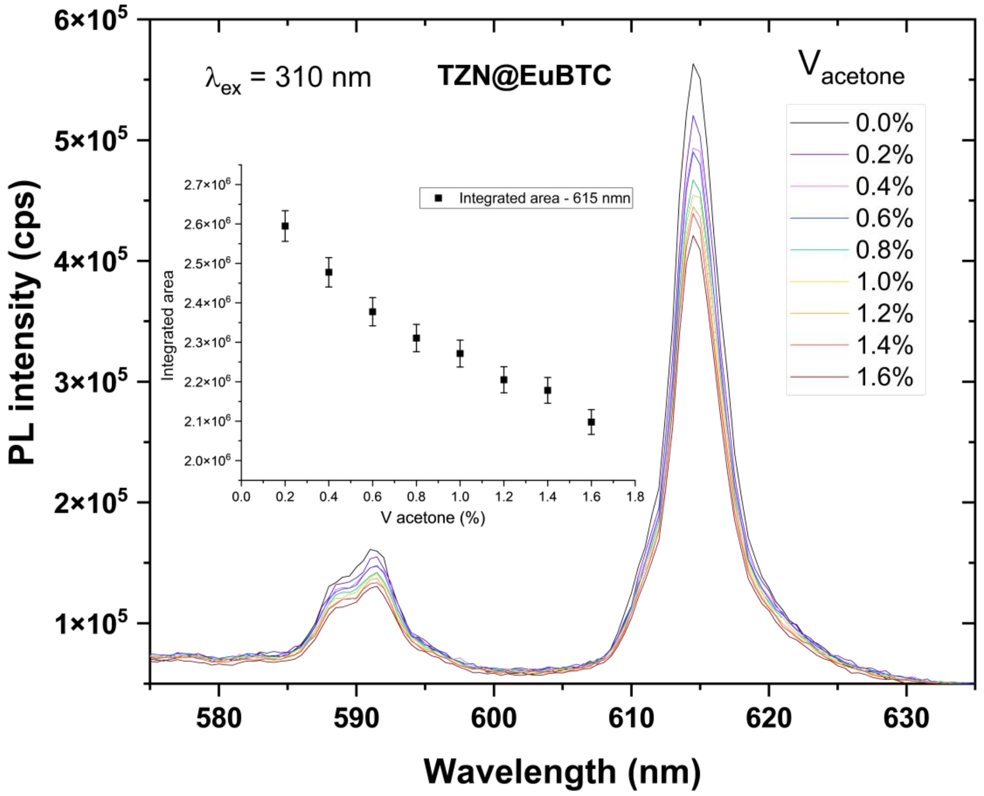

Figure S4. Linear decrease in luminescence intensity at lower concentrations of acetone, from 0.2 to 1.6%.

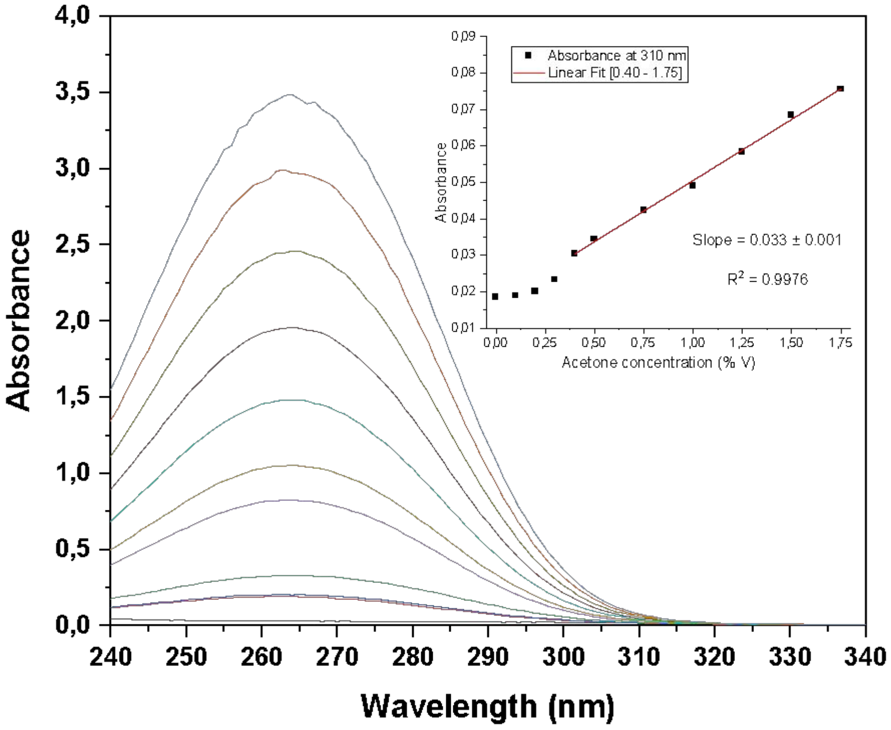

Figure S5. Absorption spectra of acetone in aqueous solution in a concentration range between 0 and 1.75% by volume. *Inset*: absorbance values at 310 nm as function of acetone concentration and linear fit from 0.40% to 1.75%.

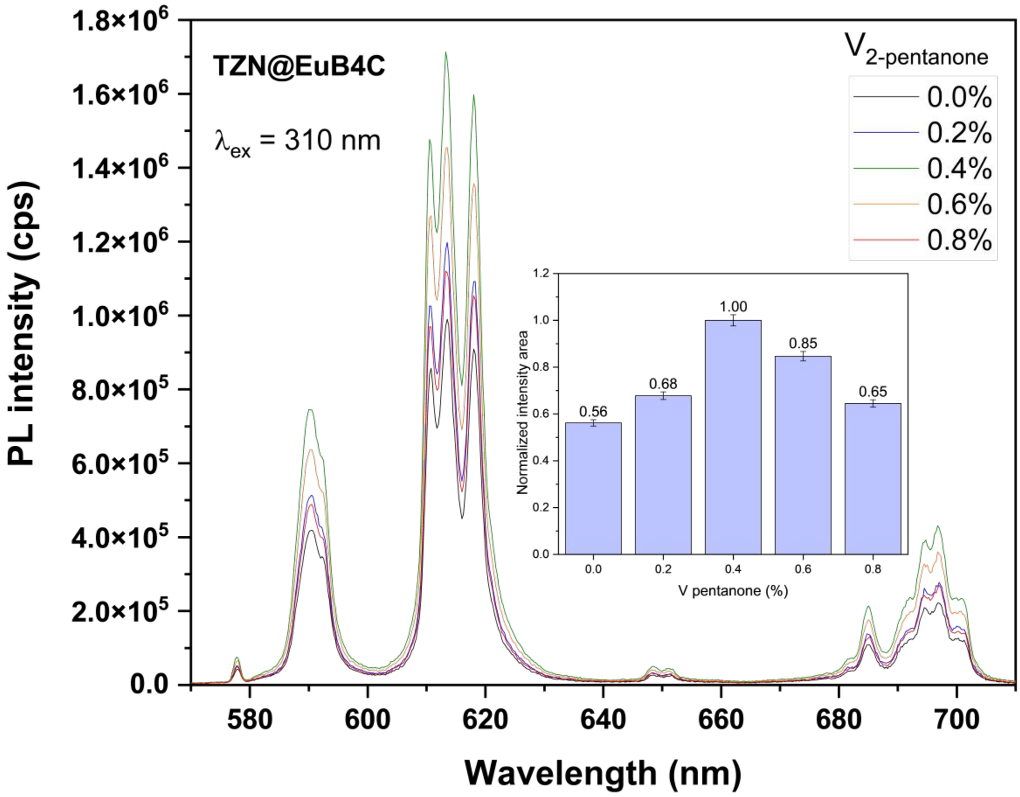

Figure S6. Luminescent response of TZN@EuB4C to low concentrations of 2-pentanone excited at 310 nm.

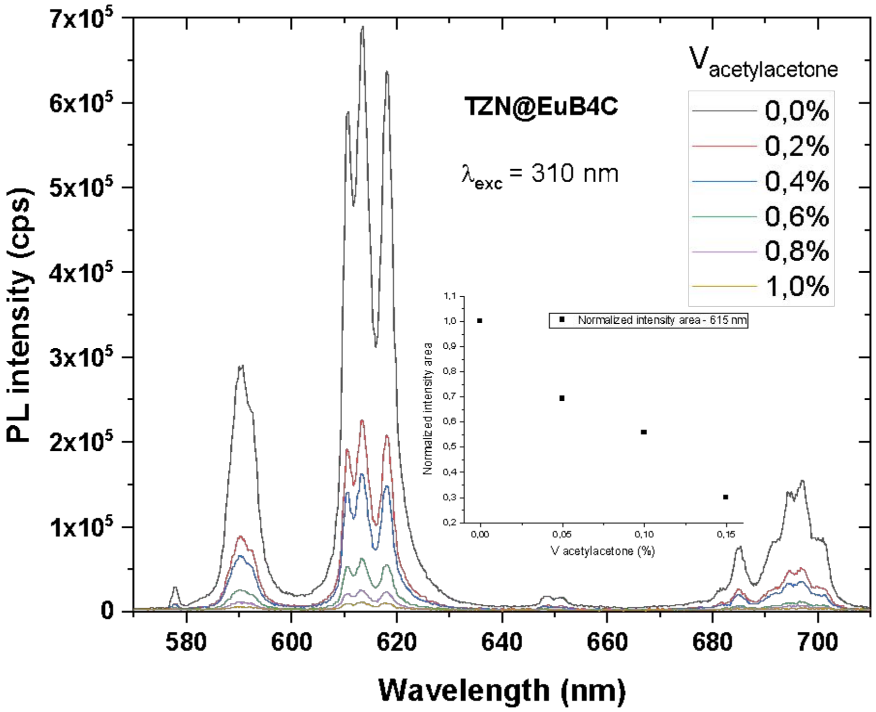

Figure S7. Luminescent response of TZN@EuB4C sample to low concentrations of acetylacetone excited at 310 nm.

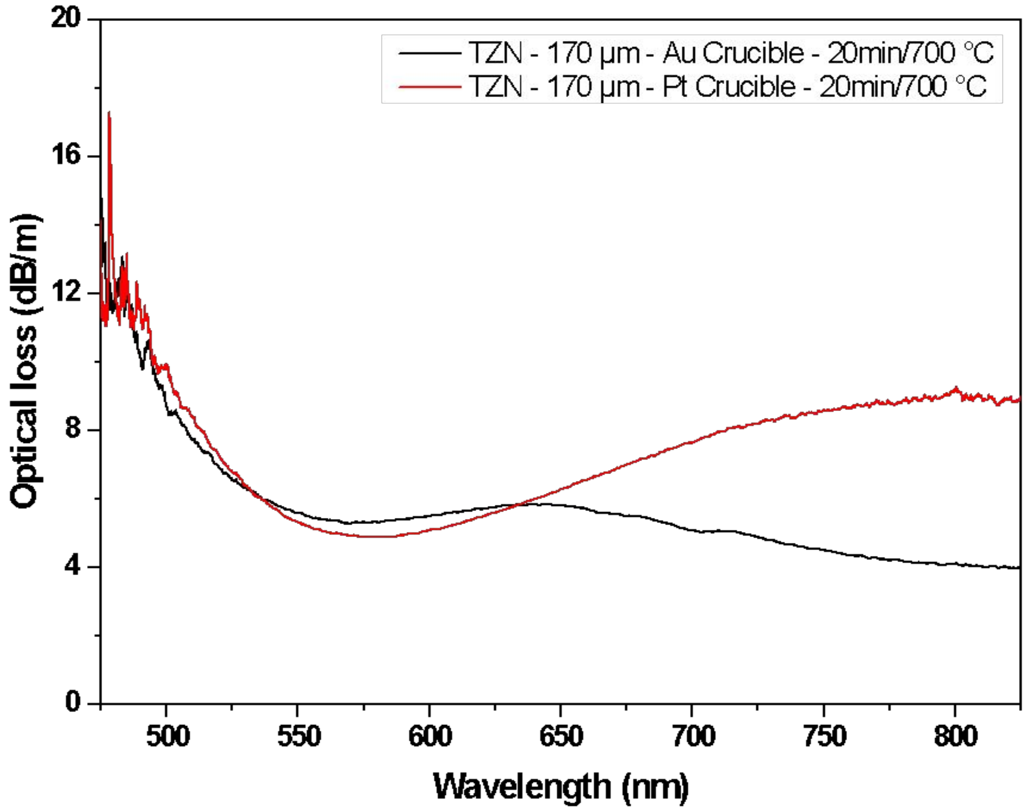

Figure S8. Attenuation curves of TZN fibers prepared in Au and Pt crucibles.

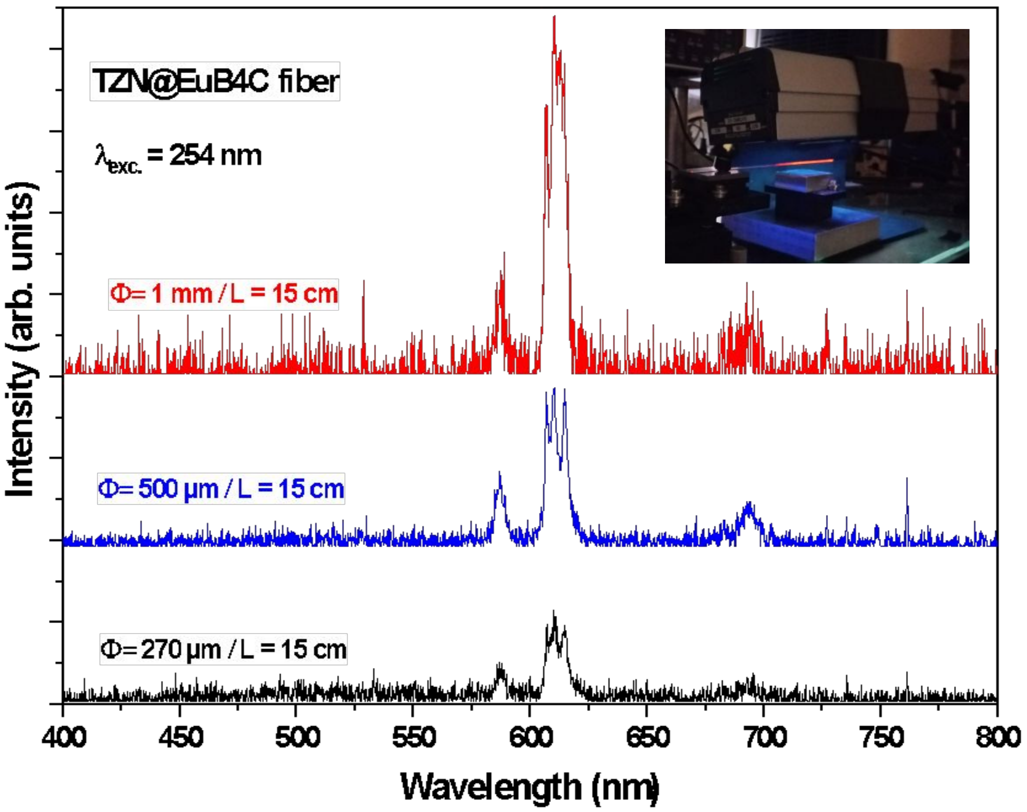

Figure S9. Luminescent spectra of TZN@EuB4C optical fibers under excitation at 254 nm. *Inset:* coated fiber illuminated by UV lamp.

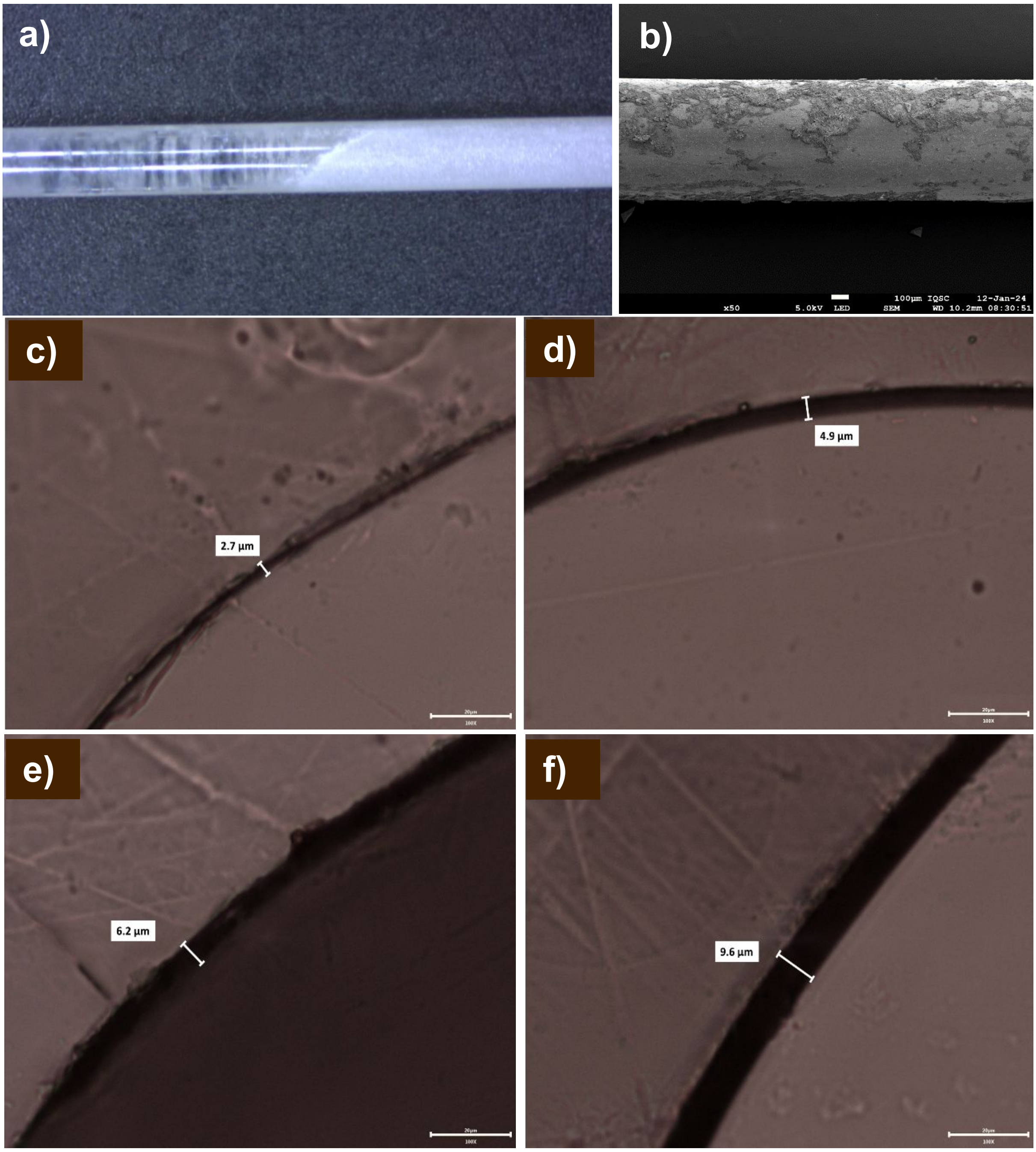

Figure S10. (a) Optical microscopy image of TZN fibers coated with EuB4C; (b) SEM image of the coated optical fiber with 81X magnification; optical microscopy images of TZN@EuB4C fibers cross-section prepared with different synthesis time: (c) 5 min; (d) 10 min; (e) 20 min; and (f) 60 min.

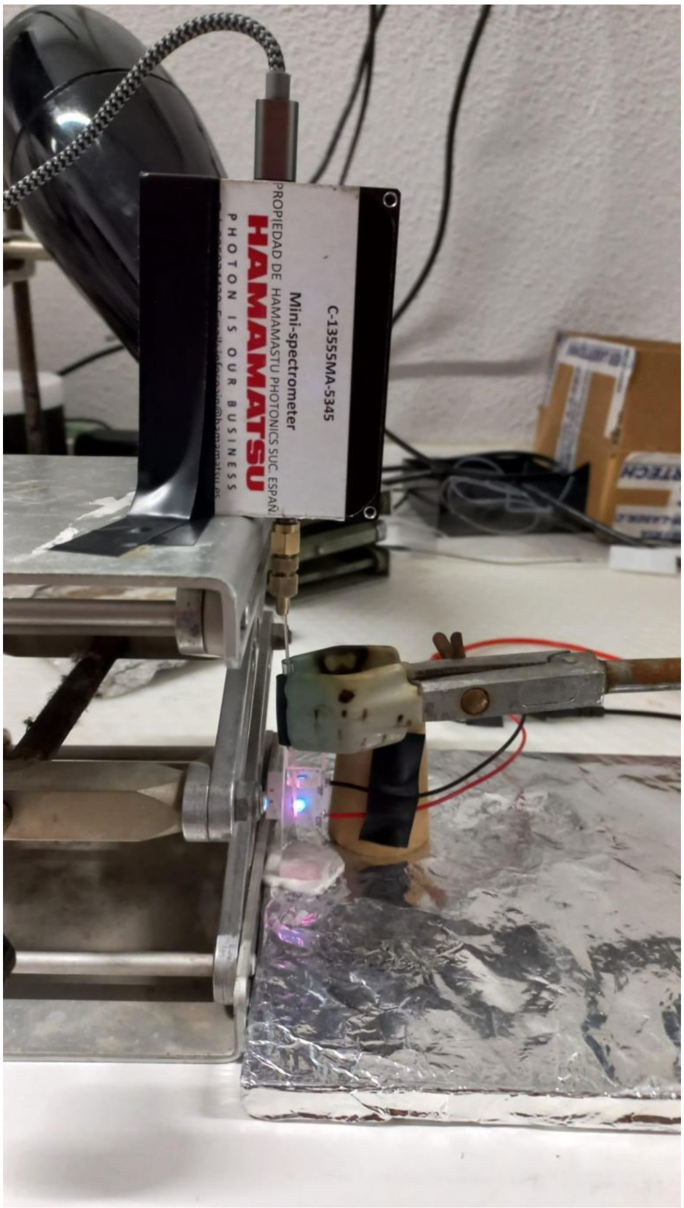

Figure S11. Experimental setup for sensing measurements using TZN optical fibers coated with Ln-CPs
